# Learning Curve Analysis of Microvascular Hepatic Artery Anastomosis for Pediatric Living Donor Liver Transplantation: Initial Experience at A Single Institution

**DOI:** 10.3389/fsurg.2022.913472

**Published:** 2022-06-17

**Authors:** Wanyi Zhou, Xiaoke Dai, Ying Le, Huiwu Xing, Bingqian Tan, Mingman Zhang

**Affiliations:** Department of Pediatric Hepatobiliary Surgery, Children’s Hospital of Chongqing Medical University, National Clinical Research Center for Child Health and Disorders, Ministry of Education Key Laboratory of Child Development and Disorders, Chongqing, China

**Keywords:** learning curve, pediatric liver transplantation, living donor, hepatic artery, anastomosis

## Abstract

**Background:**

The incidence of hepatic artery thrombosis in pediatric living donor liver transplantation (LDLT) is significantly higher than that in adults, and is closely related to the surgeon’s experience with hepatic artery anastomosis. However, there are few studies on the learning curve of hepatic artery anastomosis among surgeons.

**Methods:**

We collected data related to 75 patients who underwent pediatric LDLT and hepatic artery anastomosis independently by the same surgeon. Cumulative sum method (CUSUM) was used to analyse the duration of hepatic artery anastomosis and determine the cut-off value. Patients were divided into two phases according to CUSUM. We analysed the intraoperative and postoperative data and survival outcomes of the included patients.

**Results:**

Total anastomosis duration decreased with an increased number of completed procedures, and the average duration was 42.4 ± 2.20 min. A cut-off value and two phases were identified: 1–43 cases and 44–75 cases. Intraoperative blood loss was significantly lower in phase 2 than in phase 1. The immediate functional changes of total bilirubin (TBIL) and direct bilirubin (DBIL) were significantly also lower in phase 2 than in phase 1. Other functional outcomes, postoperative complications, and the long-term survival rate were not significantly different between the two phases.

**Conclusions:**

Technical competence in pediatric LDLT hepatic artery anastomosis may be achieved after completing 43 cases. It is a safe procedure with a surgical loupe that can be systematized and adopted by pediatric surgeons with sufficient experience via a relatively long learning curve.

## Introduction

Since the first liver transplantation (LT) was conducted by Thomas Starzl in 1963 in a 3-year-old child with biliary atresia (BA) (1), it has become the standard treatment for children with end-stage liver disease. From the first case until the early 1980s, the only technical option for pediatric LT was to transplant the entire liver of a donor with a similar weight to a recipient. Unbalanced supply–demand in organ donation was becoming increasingly prominent, particularly in children. In 1984, Bismuth et al. (2) first described a reduced-size orthotopic liver graft in pediatric LT. This surgical innovation has greatly reduced mortality in children prior to LT, and has expanded the pool of available organs in countries where cadaveric organ donation is restricted by factors such as regional culture, religious faith, and ethical issues. With refinement in technology and development of multidisciplinary management, pediatric LT has evolved considerably over the past few decades, with current long-term survival rates greater than 85% in most large-volume pediatric LT centers (3).

Although BA is the most common cause of pediatric LT (4), several new indicators such as metabolic disorders are becoming more common. However, with improved supportive treatment and medical expertise, related contraindicators are decreasing. An increasing number of transplant centers have begun LT programs in children. However, several issues remain unresolved. Hepatic artery thrombosis (HAT) is a serious complication after pediatric LT and is associated with high morbidity and mortality. The reported incidence of HAT is between 1% and 26% in children who have undergone LT (5–10). The challenge in hepatic artery anastomosis is the small diameter of the vessels (11), which requires technical acumen and good clinical judgment. The term “learning curve” is used to describe the effect of multiple repetitions resulting in an improvement of the ability to undertake a new task. The term is currently used in studies to quantify the training requirements for different surgical procedures (12, 13). However, there have been few reports defining the actual number of cases required to achieve competence in hepatic arterial anastomosis with pediatric LT.

Our center’s pediatric LT program was initiated in 2006. We conducted this study to assess improvements in operative outcomes and evaluate the applicability of a learning curve to the hepatic arterial anastomosis procedure and outcomes in pediatric LDLT.

## Materials and Methods

### Study Group

From May 2018 to June 2020, a total of 75 pediatric patients underwent living donor liver transplantation (LDLT) at the Children’s Hospital of Chongqing Medical University, Chongqing, China. All hepatic arterial anastomosis procedures were performed by the same surgeon (the second author), who initiated his own cases after following 45 cases at our center. This study was approved by the ethics committee of Chongqing Medical University and was performed in accordance with the Declaration of Helsinki of 1975, as revised in 1983. Double ethical approval was obtained from the ethical committee of Children’s Hospital of Chongqing Medical University and the ethical Committee of Human Organ Transplantation of Chongqing Health Commission for LDLT. All LDLT donors were recipients’ parents who had undergone a thorough medical, social, and psychological assessment and written informed consent was obtained in all cases.

### Preoperative Assessment

Living donors and recipients received color Doppler ultrasonography and CT angiography of the upper abdomen to evaluate the hepatic artery. The evaluation included hepatic artery imaging, variability, flow rate, diameters of the hepatic artery trunk and main branch, distribution of the intrahepatic artery, manifestation of celiac artery compression by the median arcuate ligament, and volumetric measurements of the target grafts. Whether or not there was replacement of the right or left hepatic artery should be specified in the target grafts.

### Surgical Technique

For recipients, arteries were anastomosed using standardized microsurgical techniques after venous and portal reconstruction. Arterial anastomosis was performed with a 4.3× magnification loupe (Scanlan International, Inc., St Paul, MN, USA) and 8-0 or 9-0 monofilament polypropylene sutures. According to intraoperative evaluation, the recipient artery with a diameter closest to that of the graft artery was selected, to ensure that the cutting edge was flat, without adventitial curling. Satisfactory blood flow was assessed by temporarily releasing the vascular clamp. Direct end–to–end anastomosis was performed in almost all cases. The opening can be formed into a bevel or the artery branch can be selected to form a flared mouth when there is a size discrepancy. The arterial anterior and posterior walls were located with suture placement at 0° and 180°; then, the artery was rotated 90°, and anastomosis was completed with an interrupted suture. The proximal artery could be further mobilized if the tension was too high to ensure the anastomosis was tension-free. The surgical field was irrigated with 2% lidocaine or papaverine solution whenever necessary to reduce vasospasm. The lumen was flushed with saline containing low-molecular-weight heparin sodium throughout the entire process to ensure a clear operative field and moist intima. After opening blood flow, hepatic artery pulsation and other arterial drains were observed. If these were satisfactory, other branches would not be reconstructed and could be safely ligated. An ultrasonogram was undertaken immediately to evaluate the patency of the anastomosis. The normal transplanted hepatic artery showed a pulsatile spectrum with the systolic peak velocity ≥20 cm/s, the systolic acceleration time <0.08s, and the systolic peak velocity/end diastolic velocity (S/D) value >2. Arterial steal syndrome and arcuate ligament compression syndrome were identified during the procedure, and the splenic artery and gastroduodenal artery could be ligated to increase the blood flow when necessary.

### Postoperative Management

The anticoagulation regimen was to use heparin for anticoagulation in the early stage, maintain the activated partial thromboplastin time at approximately 60 s, gradually transition to oral warfarin treatment after food intake, and adjust the dose of warfarin to maintain the international normalized ratio (INR) at 1.5–2.0. The anticoagulation duration lasted for 3–6 months. The triple immunosuppressive regimen included tacrolimus, methylprednisolone, and basiliximab; mycophenolate mofetil or cyclosporine were added when necessary. Color Doppler ultrasound was performed twice a day during week 1, once a day during week 2, 2–3 times a week during weeks 3–4, once a month from 1 to 3 months after surgery, and once every 3 months thereafter. The blood flow velocity and diameter of the hepatic vein, hepatic artery, and portal vein were monitored.

### Outcome Evaluation and Definitions

Characteristics of all patients were collected and analyzed including demographics, results of biochemical tests, indications for LDLT, history of Kasai procedure, and surgical information (blood loss, anastomosis duration), postoperative outcomes (complications, morbidity, mortality, stay in the pediatric intensive care unit (PICU), ventilation duration, hospital stay), and results of blood tests 7 days after surgery. The Clavien–Dindo classification was adapted for postoperative complications (14). Because complications of grade I and II are common in pediatric LT and usually have no serious outcomes, we only analyzed complications above grade III. The Pediatric End-Stage Liver Disease (PELD) score was calculated according to the formula: PELD score = 6.78 × ln (ALB) (g/dL) + 4.8 × ln (TBIL) (mg/dL) + 18.57 × ln (INR) + 4.36 (if the patient is less than 1 year old) + 6.43 (if the patient has growth failure) (15). The data principle of the formula is that values of ALB, TBIL, and INR less than 1.0 are set to 1.0.

### Statistical Analysis

The data and surgical characteristics of all patients were normalized using absolute counts and percentages for categorical variables and mean ± standard deviation for continuous variables. Comparisons of continuous variables were performed using an independent–samples t-test and the chi-squared or Fisher’s exact test for categorical variables. The Kaplan–Meier method with the log–rank test was used to compare long-term survival. The time from the beginning to the end of hepatic artery anastomosis was selected as the sign of completion of the procedure. All operations were sorted according to chronological sequence. The progress and evolution of hepatic artery anastomosis were evaluated using CUSUM analysis. The difference between the actual suture time of each operation and the average suture time of all cases was accumulated and summed, so as to obtain the learning curve. Statistical analysis was performed using IBM SPSS software version 24 (IBM Corp., Armonk, NY, USA), and significance was defined as *P* value <0.05.

## Results

### Learning Curve

**Figure 1:**
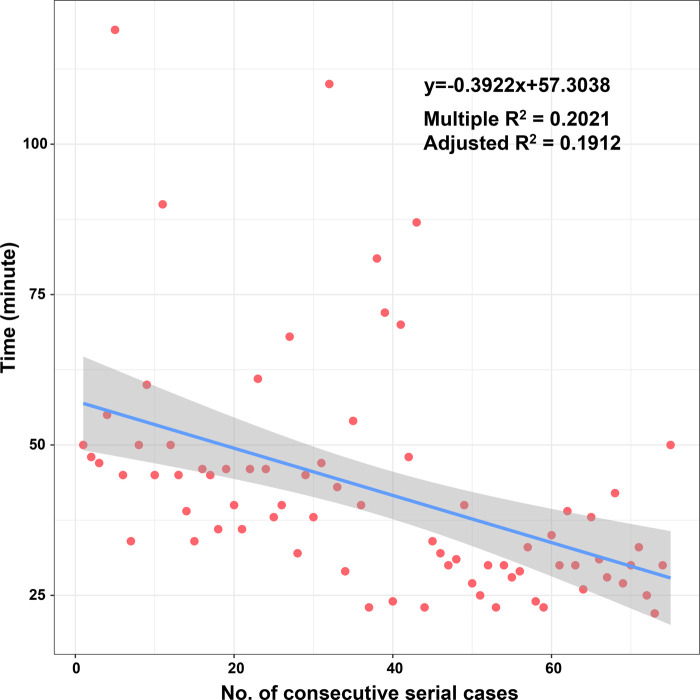
Scatter plot of operative time and progressive case number.

**Figure 2:**
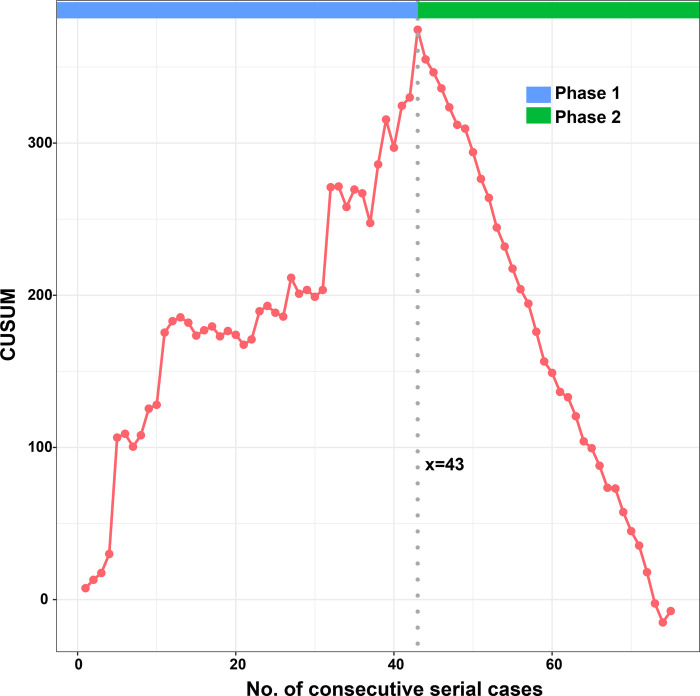
CUSUM analysis of anastomosis time.

**Figure 3:**
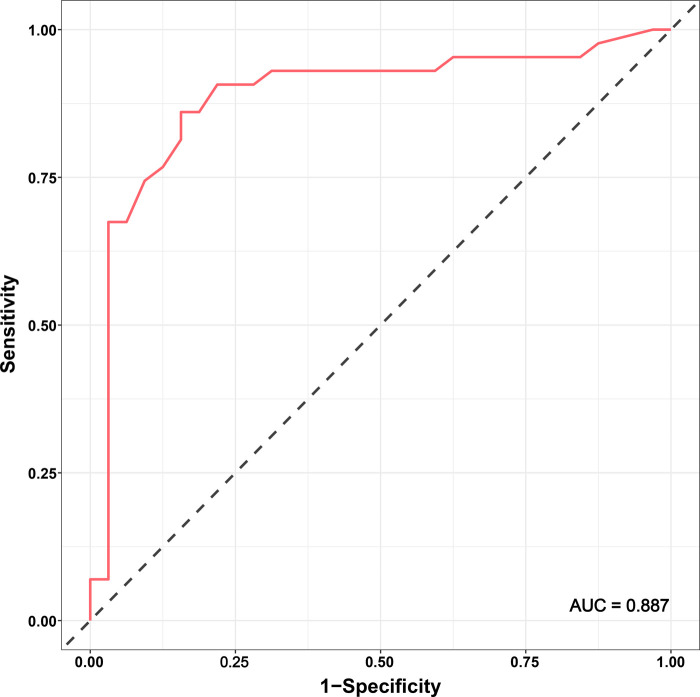
ROC curve for completion of the learning curve.

According to the linear regression model (Figure 1), anastomosis duration was significantly decreased with increased number of completed of procedures (*P* < 0.05). Concerning the two phases, CUSUM analysis showed an initial increase in anastomosis duration in phase 1, then a relatively stable median duration of procedure, and a decrease in phase 2 with an anastomosis time frequently lower than the overall mean (Figure 2). Finally, a receiver operating characteristic (ROC) curve analysis with an area under the ROC curve of 0.887 (95% confidence interval 0.819–0.971) showed that competency in hepatic anastomosis could be achieved after completing 43 procedures (Figure 3).

### Patient Characteristics and Outcomes

The characteristics of all 75 included patients are listed in Table 1. The mean age was 0.84 ± 0.16 years, and the mean graft to recipient body weight ratio (GRWR) was 3.51 ± 0.10%. Among the 75 cases, 20 had a Kasai procedure history. The indications for LDLT included BA (*n* = 68), Caroli disease (*n* = 1), urea cycle disorders (*n* = 1), Alagille syndrome (*n* = 1), congenital liver fibrosis (*n* = 2), and cavernous transformation of the portal vein (*n* = 2). The three grades of PELD score were 0–9, 10–19, and ≥20, recorded in 5 (6.7%), 37 (49.3%), and 33 (44%) cases, respectively. The median anastomosis duration was 42.4 ± 2.20 min with a median blood loss of 15.62 ± 1.48 mL/kg·min. The median PICU stay time was 174.56 ± 21.50 h, and the median ventilation duration was 74.82 ± 15.6 h. The median hospital stay was 40.59 ± 5.43 days. Six (0.8%) patients (consecutive cases 4, 17, 21, 23, 27, and 48) died: two because of respiratory failure, one because of post-transplant lymphoproliferative disease (PTLD), one owing to hemorrhage, and two because of infection. In the 75 consecutive patients, the main complications were bile leak, biliary anastomosis stricture, respiratory failure, portal vein stenosis, hemorrhage, infection, and PTLD, recorded in 3 (4%) patients, 7 (9.3%), 2 (2.7%), 17 (22.7%), 2 (2.7%), 2 (2.7%), and 1 (1.3%) patient, respectively. No hepatic artery-related complications were found in our study.

**Table 1:** Overall patients characteristics (*n *=  75).

Characteristic	Value
Age (months)	10.09 ± 16.46
Sex (F/M)	30/45
Body weight (kg)	7.45 ± 0.42
Height (cm)	64.92 ± 1.61
Blood loss (mL/kg·min)	15.62 ± 1.48
Kasai procedure	
Yes	20
No	55
Anastomosis duration (min)	42.4 ± 2.20
Indications for LDLT	
BA	68 (90.7)
Caroli disease	1 (1.3)
Urea cycle disorders	1 (1.3)
Alagille disease	1 (1.3)
Congenital liver fibrosis	2 (2.7)
CTPV	2 (2.7)
PELD score	
0–9	5 (6.7)
10–19	37 (49.3)
≥20	33 (44)
GRWR (%)	3.51 ± 0.10
Hospital stay (days)	40.59 ± 5.43
PICU stay (h)	174.56 ± 21.50
Ventilation (h)	74.82 ± 15.6
Graft type	
LLS	69 (92.0)
Left lobe without MHV	2 (2.7)
Right lobe without MHV	2 (2.7)
Left lobe with MHV	2 (2.7)
Type of complications	
Bile leak	3 (4)
Biliary anastomosis stricture	7 (9.3)
Respiratory failure	2 (2.7)
Portal vein stenosis	17 (22.7)
Haemorrhage	2 (2.7)
Infection	2 (2.7)
PTLD	1 (1.3)
HAT	0 (0)
Hepatic arterial stenosis	0 (0)
Aneurysm of the hepatic artery	0 (0)

*PELD, pediatric end–stage liver disease; GRWR, graft to recipient body weight ratio; CTPV, cavernous transformation of the portal vein; PICU, pediatric intensive care unit; LLS, left lateral segment; MHV, middle hepatic vein; PTLD, post–transplant lymphoproliferative disease; HAT, hepatic artery thrombosis.*

As shown in Table 2, there were no significant differences in terms of age, sex, body weight, height, graft artery diameter, Kasai procedure history, and PELD score between the two phases. As presented in Table 3, the main preoperative results showed that the levels of ALP and PLT in phase 1 were lower than those in phase 2 (*P* = 0.003, *P* = 0.04, respectively), and there were no significant differences in the other results between the two phases.

**Table 2:** Patients’ characteristics according to phases of the learning curve.

	Phase 1	Phase 2	*P*
Sex (F/M)	17/26	13/19	1.00
Age (months)	11.65 ± 18.32	8.00 ± 13.58	0.35
Body weight (kg)	7.73 ± 4.34	7.06 ± 2.27	0.43
Height (cm)	66.40 ± 16.33	62.94 ± 9.73	0.29
Graft artery diameter (mm)	2.73 ± 0.34	2.60 ± 0.35	0.84
Kasai procedure			
Yes	12	8	1.00
No	31	24	
PELD score			
0–9	3	2	0.94
10–19	20	17	
≥20	20	13	

**Table 3:** Patients’ preoperative results according to phases of the learning curve.

	Phase 1	Phase 2	*P*
TBIL (μmol/L)	281.22 ± 141.68	266.49 ± 131.91	0.65
DBIL (μmol/L)	220.15 ± 117.38	197.05 ± 95.43	0.37
Alb (g/L)	37.01 ± 6.49	35.87 ± 6.45	0.45
ALT (U/L)	215.48 ± 158.32	191.59 ± 71.84	0.43
AST (U/L)	398.56 ± 309.37	393.50 ± 217.47	0.94
ALP (U/L)	535.50 ± 282.03	785.20 ± 412.65	0.003
GGT (U/L)	380.94 ± 361.66	266.05 ± 244.81	0.13
CREA (μmol/L)	16.75 ± 7.07	15.11 ± 4.72	0.26
INR	1.39 ± 0.65	1.31 ± 0.28	0.51
PLT (*10^9^/L)	209.44 ± 101.17	265.09 ± 128.18	0.04
HGB (g/L)	97.02 ± 15.94	97.41 ± 15.58	0.92

*ALB, albumin; ALP, alkaline phosphatase; ALT, alanine aminotransferase; AST, aspartate aminotransferase; CREA, creatinine; DBIL, direct bilirubin; HGB, hemoglobin; INR, international normalized ratio; PLT, platelet; TBIL, total bilirubin.*

**Figure 4:**
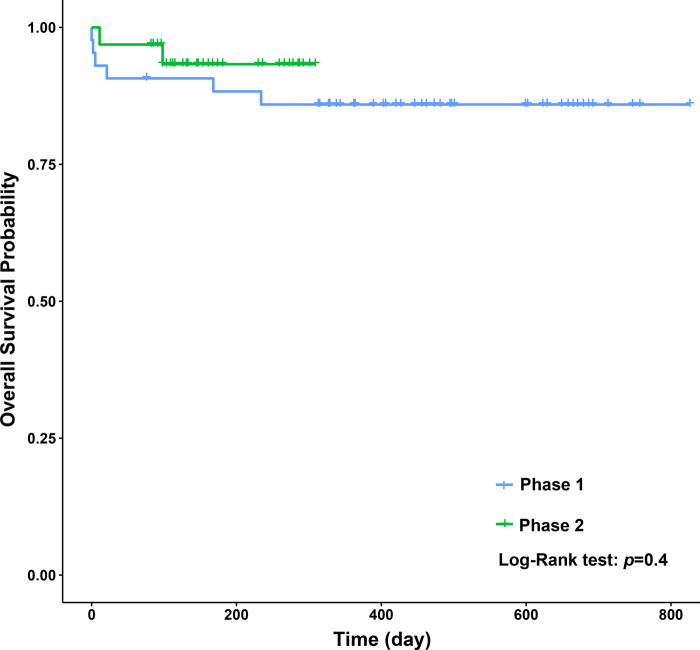
Overall patients survival (Kaplan–Meier analysis).

When we compared the operative and postoperative results of the two phases (Table 4), the blood loss in phase 2 was dramatically lower than that in phase 1. As for the blood test results 7 days after surgery, the levels of TBIL and DBIL in phase 2 were lower than those in phase 1 (*P* = 0.02, *P* = 0.05, respectively), and the levels of ALP and PLT in phase 1 were lower than those in phase 2 (*P* = 0.01, *P* = 0.00, respectively). No significant differences were observed in GRWR, ventilation time, PICU stay, hospital stay, complications, and other blood test results. Concerning long-term survival rates, no significant differences were observed between the two phases (Figure 4).

**Table 4:** Patients’ operative and postoperative outcome according to phases of the learning curve.

	Phase 1	Phase 2	*P*
Anastomosis duration (min)	51.20 ± 20.55	30.56 ± 6.18	0.00
Blood loss (mL/kg·min)	20.36 ± 14.19	9.26 ± 6.68	0.00
GRWR (%)	3.56 ± 0.92	3.44 ± 0.85	0.57
Ventilation (h)	86.85 ± 169.61	58.65 ± 64.29	0.38
PICU stay (h)	193.27 ± 233.52	149.42 ± 97.97	0.32
Hospital stay (d)	34.76 ± 18.04	48.40 ± 68.70	0.21
TBIL (μmol/L)	42.89 ± 47.48	22.49 ± 15.34	0.02
DBIL (μmol/L)	22.20 ± 34.80	9.41 ± 11.61	0.05
Alb (g/L)	48.42 ± 6.53	49.17 ± 6.22	0.62
ALT (U/L)	146.92 ± 108.18	151.54 ± 120.53	0.86
AST (U/L)	81.54 ± 107.43	79.51 ± 101.03	0.94
ALP (U/L)	67.31 ± 32.59	91.60 ± 49.84	0.01
GGT (U/L)	109.34 ± 146.61	101.11 ± 93.80	0.78
CREA (μmol/L)	21.21 ± 10.81	19.13 ± 13.99	0.47
INR	1.59 ± 0.45	1.58 ± 0.45	0.98
PLT (*10^9^/L)	125.93 ± 67.49	224.90 ± 102.20	0.00
HGB (g/L)	99.11 ± 15.23	97.37 ± 12.79	0.60
Type of complications			
Bile leak	1 (2.3)	2 (6.25)	
Biliary anastomosis stricture	3 (6.98)	4 (12.5)	
Respiratory failure	2 (4.65)	0 (0)	
Portal vein stenosis	14 (32.6)	3 (9.4)	
Haemorrhage	1 (2.3)	1 (3.12)	
Infection	0 (0)	2 (6.25)	
PTLD	1 (2.3)	0 (0)	
HAT	0 (0)	0 (0)	
Hepatic arterial stenosis	0 (0)	0 (0)	
Aneurysm of the hepatic artery	0 (0)	0 (0)	
Total	22 (51.1)	12 (37.5)	0.48

## Discussion

LT is the only effective method for treating end-stage liver disease. In recent years, with innovations in surgical technology and the development of multidisciplinary collaborative therapy, the use of pediatric LT has flourished. In 10 years, the rate of pediatric LT in China has increased from 1%–4% to approximately 20%. In 2017, a total of 722 pediatric LTs were completed in mainland China (China Liver Transplant Registry [CLTR] data), which was a milestone year. Arterial reconstruction is a crucial step in LT especially because graft arteries from living donors are shorter and smaller than those from deceased donors. Importantly, children should never be considered a smaller version of an adult. Additionally, there is a higher incidence of technical complications in pediatric LT (16). Early experiences in pediatric LDLT are plagued by vascular complications, with rates of arterial thrombosis as high as 28% (17). Moreover, there is no fixed pattern for hepatic artery anastomosis, and the surgeon must determine the preferred method and have sufficient expertise to successfully complete the procedure.

Herein, we present a process of gradual evolution of a surgeon in performing microvascular hepatic artery anastomosis for pediatric LDLT in the form of a learning curve. We used the CUSUM method to normalize values against the mean and summed the differences to demonstrate the trend and a change point. Although this method is not standard for qualification judgment, it is recommended as an effective tool (18).

From the linear regression model fit to the data, a significant decrease in operative time can be observed. The learning curve indicates that it is necessary to complete 43 cases to reach competence and proficiency. The second phase of LDLT illustrates that after an initial period of practice, the surgeon has improved his technical skills and systematized the procedure.

In this study, our main purpose was to use the anastomosis duration to analyze improvement in the results within a period after the start of hepatic artery anastomosis; the secondary purpose was to evaluate the recipient’s postoperative outcome. We therefore analyzed the effect of the learning curve on patients’ immediate outcomes and long-term survival rates in the two phases. We observed that the learning curve had an influence on blood loss during the procedure and the levels of TBIL and DBIL on day 7 after surgery. However, considering the correlation between intraoperative blood loss and overall operative time, this may not be a direct result of the reduction in hepatic artery anastomosis time. Not only was the rate of complications similar between the two groups, the functional recovery processes and long-term survival rates were also similar. This is in keeping with the results of previous studies on the learning curve in adult LDLT (19, 20). These results might encourage some centers to initiate a pediatric LDLT program. Besides the cumulative experience, an important point is whether there is an association between low case volume and poor outcomes in pediatric LDLT. One study showed inferior outcomes in pediatric LT associated with low-volume centers (21). In addition to the effect of the learning curve of a single surgeon, we should recognize that pediatric LDLT is a process of joint learning and improvement as part of a multidisciplinary medical team. Our center performed deceased donor LT (DDLT) for several years before we initiated the LDLT program, and we received guidance from other more experienced centers. In our previous study, the surgeon who performed this procedure had considerable clinical experience in pediatric hepatobiliary surgery and was under the supervision of other experts at the beginning of his training. Moreover, although no hepatic artery complications were found in the present study, a previous study at our center showed that the incidence of hepatic artery complications in the DDLT group was 21.2%, which is consistent with reports from other centers (22, 23).

In the research on hepatic artery anastomosis in pediatric LDLT, some publications have focused on differences in the use of a microscope and a surgical loupe. There is a major concern regarding a purse-string effect when performing continuous suturing using a surgical loupe (24). However, large-sized equipment and the long period from experimental training to clinical operation with a microscope have led some centers to consult with microsurgeons when performing hepatic artery anastomosis in LDLT. Meanwhile, there are increasing reports of equivalent results using a loupe instead of a microscope (25, 26). Among our center’s early cases, there was indeed a higher incidence of complications with continuous suturing using a surgical loupe (14.9%). The surgeon was also continuously improving and practicing on the rat artery and hepatic artery simulation tool to modify his anastomosis method. After the surgeon changed the suture method and improved the suture technique, complications of the hepatic artery decreased significantly.

Reducing technical complications of the hepatic artery can significantly improve the efficacy of LT and is an important way to increase the survival rate in pediatric LT (27). Paying greater attention to evaluation of the hepatic artery and remaining up-to-date on the latest anastomosis techniques are important measures to reduce technical complications of the hepatic artery. Our center has performed detailed research on hepatic artery evaluation, and we have accumulated valuable experience. We consider that the anatomy of the hepatic artery is an important part of preoperative evaluation. Evaluation of the hepatic artery by the surgeon should start with the selection of liver transplant donor and recipient and run through the entire LT process. Fully detailed evaluation of the hepatic artery, an optimized hepatic artery anastomosis plan, and appropriate treatment skills can compensate for the risk of hepatic artery complications caused by anatomical factors.

There are several limitations in the present study. First, mainly due to the relatively small sample size and a single surgeon’s experience, which definitely induce subjectivity, our findings are not applicable to all cases. Second, our findings were largely dependent on completed medical records in this retrospective study. However, to the best of our knowledge, this is the first report of a single-center experience regarding the learning curve for hepatic artery reconstruction focusing on pediatric LDLT.

In conclusion, hepatic artery anastomosis using a surgical loupe in pediatric LDLT is a safe procedure when conducted by an experienced pediatric surgeon. The learning curve essentially represents the accumulation of experience. Our results suggest that a surgeon can obtain technical competency and proficiency after completing 43 cases. In our study, the learning curve affected intraoperative blood loss and had little influence on the immediate functional results after surgery; additionally, it had no effect on postoperative complications and the long-term survival rate. The authors recommend having a dedicated transplant team with professional guidance at the beginning of a new program to achieve better outcomes. Further study of the learning curve of other surgeons will be required to evaluate bias introduced by personal and institutional factors.

## Data Availability

The raw data supporting the conclusions of this article will be made available by the authors, without undue reservation.

## References

[1] Starzl TE Marchioro TL Vonkaulla KN Hermann G Brittain RS Waddell WR. Homotransplantation of the liver in humans. Surg Gynecol Obstet. (1963) 117:659–76. 10.1016/j.transproceed.2006.02.120 14100514 PMC2634660

[2] Bismuth H Houssin D. Reduced-sized orthotopic liver graft in hepatic transplantation in children. Surgery. (1984) 95(3):367–70. 10.1007/978-94-009-5018-4_22 6367125

[3] Lauterio A Sandro SD Concone G Carlis RD Giacomoni A Carlis LD. Current status and perspectives in split liver transplantation. World J Gastroenterol. (2015) 39:78–90. 10.3748/wjg.v21.i39.11003 PMC4607900 26494957

[4] Sundaram SS Mack CL Feldman AG Sokol RJ. Biliary atresia: indications and timing of liver transplantation and optimization of pre-transplant care. Liver Transpl. (2017) 23(1):96–109. 10.1002/lt.24640 27650268 PMC5177506

[5] Baerg J Zuppan C Klooster M. Biliary atresia–a fifteen-year review of clinical and pathologic factors associated with liver transplantation. J Pediatr Surg. (2004) 39(6):800–3. 10.1016/j.jpedsurg.2004.02.020 15185199

[6] Broering DC Kim JS Mueller T Fischer L Ganschow R Bicak T One hundred thirty-two consecutive pediatric liver transplants without hospital mortality. Ann Surg. (2004) 240(6):1002–12. 10.1097/01.sla.0000146148.01586.72 15570206 PMC1356516

[7] Lallier M Stvil D Dubois J Paradis K Laberge JM Bensoussan A Vascular complications after pediatric liver transplantation. J Pediatr Surg. (1995) 30(8):1122–6. 10.1016/0022-3468(95)90002-0. 7472963

[8] Mazzaferro V Esquivel CO Makowka L Belle S Kahn D Koneru B Hepatic artery thrombosis after pediatric liver transplantation–a medical or surgical event? Transplantation. (1989) 47(6):971–7. 10.1097/00007890-198906000-00011 2472028

[9] Mazzaferro V Esquivel CO Makowka L Kahn D Scotti-Foglieni CL. Factors responsible for hepatic artery thrombosis after pediatric liver transplantation. Transplant Proc. (1989) 21(1 Pt 2):2466–7. 10.1177/00220345900690100601 2652807 PMC2966154

[10] Tiao GM Alonso MH Ryckman FC. Pediatric liver transplantation. Semin Pediatr Surg. (2006) 15(3):218–27. 10.1053/j.sempedsurg.2006.03.008 16818143

[11] Sanada Y Mizuta K Urahashi T Umehara M Wakiya T Okada N Pediatric living donor liver transplantation using liver allograft with hemangioma. Ann Transplant Q Pol Transplant Soc. (2011) 16(1):66–9. 10.1111/j.1600-6143.2010.03354.xv21.i39.11003 21436777

[12] Gonzalvo A Fitt G Liew S de la Harpe D Turner P Ton L The learning curve of pedicle screw placement: how many screws are enough? Spine. (2009) 34(21):E761–5. 10.1097/BRS.0b013e3181b2f928 19934796

[13] Pernar LIM Robertson FC Tavakkoli A Sheu EG Brooks DC Smink DS. An appraisal of the learning curve in robotic general surgery. Surg Endosc. (2017) 31(11):4583–96. 10.1007/s00464-017-5520-2 28411345

[14] Clavien PA Camargo CA Jr. Croxford R Langer B Levy GA Greig PD. Definition and classification of negative outcomes in solid organ transplantation. Application in liver transplantation. Ann Surg. (1994) 220(2):109–20. 10.1097/00000658-199408000-00002 8053733 PMC1234350

[15] Bourdeaux C Tri TT Gras J Sokal E Otte JB de Goyet JD PELD score and posttransplant outcome in pediatric liver transplantation: a retrospective study of 100 recipients. Transplantation. (2005) 79(9):1273–6. 10.1097/00007890-200505150-00060 15880084

[16] Bekker J Ploem S de Jong KP. Early hepatic artery thrombosis after liver transplantation: a systematic review of the incidence, outcome and risk factors. Am J Transplant. (2009) 9(4):746–57. 10.1111/j.1600-6143.2008.02541.x 19298450

[17] Mori K Nagata I Yamagata S Sasaki H Nishizawa F Takada Y The introduction of microvascular surgery to hepatic artery reconstruction in living-donor liver transplantation–its surgical advantages compared with conventional procedures. Transplantation. (1992) 54(2):263–8. 10.1097/00007890-199208000-00014 1496539

[18] Berardi G Aghayan D Fretland Å A Elberm H Cipriani F Spagnoli A Multicentre analysis of the learning curve for laparoscopic liver resection of the posterosuperior segments. Br J Surg. (2019) 106(11):1512–22. 10.1002/bjs.11286 31441944

[19] Li C Mi K Wen T Yan L Li B Yang J A learning curve for living donor liver transplantation. Dig Liver Dis. (2012) 44(7):597–602. 10.1016/j.dld.2012.01.016 22387283

[20] Kim SH Cho SY Park SJ Lee KW Han SS Lee SA Learning curve for living-donor liver transplantation in a fledgling cancer center. Transpl Int. (2009) 22(12):1164–71. 10.1111/j.1432-2277.2009.00934.x 19891045

[21] Tracy ET Bennett KM Danko ME Diesen DL Westmoreland TJ Kuo PC Low volume is associated with worse patient outcomes for pediatric liver transplant centers. J Pediatr Surg. (2010) 45(1):108–13. 10.1016/j.jpedsurg.2009.10.018 20105589

[22] Feng MX Zhang JX Wan P Qiu BJ Gu LH Zhang JJ Hepatic artery reconstruction in pediatric liver transplantation: Experience from a single group. Hepatobiliary Pancreat Dis Int. (2020) 19(4):307–10. 10.1016/j.hbpd.2020.06.014 32690249

[23] Orlandini M Feier FH Jaeger B Kieling C Vieira SG Zanotelli ML. Frequency of and factors associated with vascular complications after pediatric liver transplantation. J Pediatr. (2014) 90(2):169–75. 10.1016/j.jped.2013.08.010 24370174

[24] Song S Kwon CH Moon HH Lee S Kim JM Joh JW Single-center experience of consecutive 522 cases of hepatic artery anastomosis in living-donor liver transplantation. Transplant Proc. (2015) 47(6):1905–11. 10.1016/j.transproceed.2015.06.014 26293071

[25] Balci D Ahn CS. Hepatic artery reconstruction in living donor liver transplantation. Curr Opin Organ Transplant. (2019) 24(5):631–6. 10.1097/MOT.0000000000000697 31483339

[26] Li PC Thorat A Jeng LB Yang HR Li ML Yeh CC Hepatic artery reconstruction in living donor liver transplantation using surgical loupes: achieving low rate of hepatic arterial thrombosis in 741 consecutive recipients-tips and tricks to overcome the poor hepatic arterial flow. Liver Transpl. (2017) 23(7):887–98. 10.1002/lt.24775 28422392

[27] Enne M Pacheco-Moreira L Balbi E Cerqueira A Alves J Valladares MA Hepatic artery reconstruction in pediatric living donor liver transplantation under 10 kg, without microscope use. Pediatr Transplant. (2010) 14(1):48–51. 10.1111/j.1399-3046.2009.01219.x 19656321

